# Mechanisms outpacing medicine in autoinflammatory diseases

**DOI:** 10.3389/fimmu.2026.1868804

**Published:** 2026-06-17

**Authors:** Ruyue Chen

**Affiliations:** Department of Nephrology and Immunology, Children’s Hospital of Soochow University, Suzhou, China

**Keywords:** autoinflammation, inflammasome, interferonopathy, monogenic autoinflammatory disease, NF-κB, targeted therapy

## Abstract

Autoinflammatory diseases are increasingly defined at the genetic and pathway levels, yet therapeutic predictability has not advanced in parallel. This disconnect is especially evident in monogenic disorders, in which molecular diagnosis can identify the initiating lesion but does not reliably predict the dominant effector process, organ-specific risk, or the treatment most likely to achieve durable control. Similar clinical phenotypes may arise from distinct molecular defects, whereas disruption of the same pathway, or even the same gene, may produce divergent organ involvement, disease evolution, and therapeutic response. In established disease, the initiating lesion, sustaining inflammatory circuitry, and the process currently driving tissue injury may no longer remain aligned. This review examines how that misalignment complicates therapeutic decision-making in autoinflammation, with emphasis on type I interferonopathies, inflammasome-related disorders, and NF-κB-centered diseases. It argues that treatment should be genotype-informed rather than genotype-determined, integrating the current dominant inflammatory module, organ-prioritized disease burden, disease stage, tissue reversibility, and treatment-limiting complications. A framework that is phenotype-weighted, organ-prioritized, and time-sensitive may better align mechanism-based therapy with real-world clinical outcomes.

## 1 Introduction

Autoinflammation entered medicine as a clinical observation before it became a mechanistic category. Familial Mediterranean fever (FMF) had long been recognized as a recurrent inflammatory disorder, but the field moved decisively into the molecular era with the identification of *MEFV* in 1997 (1, 2). This was followed by the discovery of *TNFRSF1A/TNFR1* mutations in TNF receptor-associated periodic syndrome (TRAPS) and *MVK* mutations in mevalonate kinase deficiency/hyper-IgD syndrome (MKD/HIDS) in 1999, establishing that clinically similar inflammatory phenotypes could arise from distinct genetic lesions (3, 4). Subsequent identification of *CIAS1/NLRP3* mutations in familial cold autoinflammatory syndrome (FCAS) and Muckle-Wells syndrome (MWS) in 2001, and in chronic infantile neurologic, cutaneous, and articular (CINCA) syndrome in 2002, further unified apparently separate entities into a shared cryopyrin-associated disease continuum (5, 6). These advances culminated in the concept of autoinflammatory disease as inflammation arising in the absence of high-titer autoantibodies or antigen-specific T cells, thereby distinguishing these disorders from classical autoimmune diseases dominated by adaptive immune dysregulation (7). This distinction was later refined by recognition that autoinflammatory and autoimmune mechanisms are not mutually exclusive, but frequently coexist across a continuum of self-directed inflammatory disease (8).

Over the past two decades, the autoinflammatory framework has expanded far beyond hereditary periodic fever syndromes to include type I interferonopathies, inflammasome-related disorders, disorders of NF-κB and ubiquitin signaling, and acquired conditions such as VEXAS syndrome (9–12). This shift from syndrome-based recognition to pathway-based classification has sharpened disease definition and highlighted therapeutically relevant inflammatory nodes. It has also exposed a central clinical paradox: similar phenotypes may arise from distinct lesions, whereas disruption of the same pathway, or even the same gene, can produce markedly different organ involvement, disease trajectories, and treatment response (13–18). Monogenic autoinflammatory diseases seldom follow a linear gene-to-pathway-to-therapy model; instead, they behave as multilayered inflammatory systems in which the initiating lesion, dominant effector circuitry, and most actionable therapeutic target may separate over time (9, 17–19). Phenotypic overlap can reflect convergence on shared downstream modules (9, 13), whereas divergence within the same gene may reflect allele-specific effects, modifier background, somatic mosaicism, and environmental context (20, 21). Recent data from coatomer protein α (COPA) syndrome further substantiate this modifier framework, showing that the common HAQ allele of STING can attenuate COPA-dependent STING signaling and modify clinical penetrance in carriers of pathogenic COPA variants (22). Related observations at *TNFAIP3*/A20 and *IFIH1*/MDA5 extend this concept toward a monogenic-polygenic continuum, in which common variants shape susceptibility to complex autoimmunity, whereas rare high-impact variants in the same genes or pathways can drive monogenic immune dysregulation (23–26). As disease evolves, the mechanisms that initiate inflammation may no longer be those that sustain tissue injury. Treatment response is therefore shaped not only by genotype but also by organ involvement, disease stage, tissue reversibility, and parallel inflammatory circuits (18, 27). This review uses monogenic autoinflammatory diseases as a human model to examine why mechanism-based therapy often fails to yield durable clinical control and proposes a framework that is genotype-informed, phenotype-weighted, organ-prioritized, and time-sensitive.

## 2 Similar phenotypes, different mechanisms

### 2.1 Phenotypic overlap in autoinflammation

**Figure 1:**
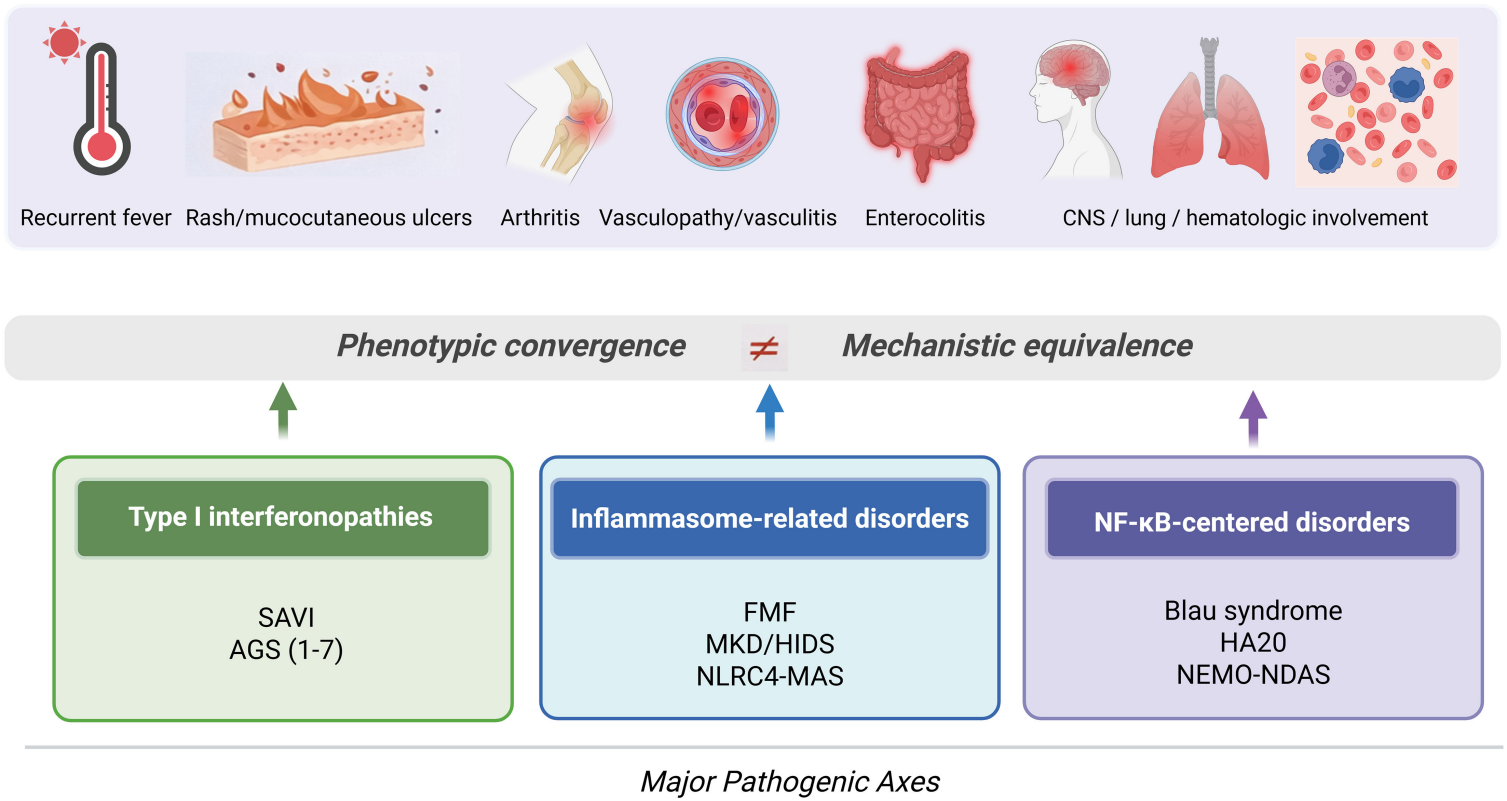
Similar phenotypes across distinct pathogenic axes in autoinflammatory diseases. Clinically overlapping manifestations, including recurrent fever, rash or mucocutaneous ulceration, arthritis, vasculopathy or vasculitis, enterocolitis, and central nervous system, pulmonary, or hematologic involvement, may arise from distinct pathogenic axes in autoinflammatory diseases. Representative disorders are organized according to three major mechanistic categories: type I interferonopathies, inflammasome-related disorders, and NF-κB-centered disorders. This schematic illustrates representative patterns of phenotypic convergence across these axes and emphasizes that clinical similarity does not necessarily indicate mechanistic equivalence or identical therapeutic dependency.

Systemic autoinflammatory diseases are often clinically recognizable, but phenotypically imprecise. Across genetically distinct disorders, recurrent or persistent sterile inflammation converges on a limited repertoire of shared manifestations (13, 28). Fever remains the most common and informative presenting feature, particularly in childhood-onset disease, and recurrent fever without an infectious explanation is often the main entry point to diagnosis (9, 29, 30). Many patients initially present with stereotyped febrile episodes, as in periodic fever with aphthous stomatitis, pharyngitis, and adenitis (PFAPA) syndrome and hereditary recurrent fever disorders such as FMF, TRAPS, and MKD/HIDS (29, 31). Beyond fever, cutaneous and mucocutaneous inflammation, musculoskeletal symptoms, serositis, vasculopathic or vasculitic lesions, and gastrointestinal involvement recur across multiple entities rather than defining discrete clinical categories (9, 32). (**Figure 1**) Certain features may sharpen suspicion without being disease-defining: urticaria-like rash with sensorineural hearing loss may point toward cryopyrin-associated disease (33, 34), vasculitic and vasculopathic skin lesions may suggest interferon-driven disease (19, 35), and early-onset bowel inflammation or Behçet-like features may raise suspicion for NF-κB-related disorders (36, 37). Yet such clues rarely permit confident mechanistic inference. At presentation, the fundamental problem is therefore one of cross-sectional overlap: distinct molecular defects repeatedly generate similar inflammatory phenotypes, and phenotype alone seldom provides sufficient specificity for mechanistic inference or definitive classification.

Phenotypic overlap is also longitudinal. Early manifestations are often incomplete, nonspecific, or deceptively mild, whereas more informative features emerge only as disease evolves. Later-onset Aicardi-Goutières syndrome (AGS), particularly in association with *RNASEH2B*, may initially present with sterile pyrexias and irritability before progressing to a more recognizable neurodevelopmental phenotype with developmental delay or regression, acquired microcephaly, spasticity, and truncal hypotonia (38, 39). Deficiency of adenosine deaminase 2 (DADA2) may begin with recurrent fever or constitutional inflammation before progressing into livedo racemosa, polyarteritis nodosa-like vascular disease, gastrointestinal involvement, or stroke (40–42). Haploinsufficiency of A20 (HA20) may likewise begin with fever, rash, or intestinal inflammation before a more recognizable Behçet-like phenotype with oral or genital ulceration and multisystem inflammation becomes apparent (43–45). Age at onset further shapes interpretation without materially improving specificity. Childhood-onset disease more often enters practice through recurrent febrile syndromes (29, 46), whereas adult-onset disease is more likely to be framed initially within the broader differential diagnosis of vasculitis, inflammatory bowel disease, Still’s disease, or Behçet-like disease (9, 32, 47, 48). Clinical labels are therefore often provisional early in the disease course. Phenotypic overlap is not merely a diagnostic inconvenience; it is a fundamental reason why clinical presentation alone cannot reliably disclose mechanism or support confident therapeutic inference at the bedside.

### 2.2 Pathogenic heterogeneity in autoinflammation

Shared phenotypes in autoinflammation do not imply shared mechanism. In the 2024 IUIS framework, monogenic autoinflammatory disorders are grouped into type I interferonopathies, inflammasome-related disorders, and non-inflammasome conditions (21). For clinical practice, this heterogeneity matters at three levels: different pathways may converge on similar inflammatory phenotypes; a single pathway can produce strikingly different organ-predominant disease; and even within one gene, allelic effects and disease evolution can reshape therapeutic dependency (**Table 1**). Type I interferonopathies illustrate how a shared pathogenic axis can still generate highly heterogeneous clinical disease. This category, initially centered on AGS, expanded as apparently unrelated Mendelian disorders were recognized to share a backbone of inappropriate type I interferon activation (49, 50). It now includes STING-associated vasculopathy with onset in infancy (SAVI), pediatric systemic lupus erythematosus (SLE) due to DNASE1L3 deficiency, spondyloenchondrodysplasia with immune dysregulation (SPENCD), and other related disorders (19). Although unified by excessive type I interferon activity, these conditions span variable combinations of neuroinflammation, vasculopathy, inflammatory skin disease, interstitial lung disease, lipodystrophy, and systemic inflammation (19, 50–52). Such divergence likely reflects differences in the upstream lesion, dominant cellular and tissue targets, timing and persistence of interferon activation, compensatory pathway usage, variant-specific molecular consequences, and modifying genetic and environmental context (52). Thus, even when the broad pathway is known, the clinically dominant organ phenotype and the most actionable therapeutic dependency may still differ substantially across patients. In the 2024 IUIS genotypic classification, this category comprises 23 disease entities associated with 21 genetic defects (21).

**Table 1:** Representative monogenic autoinflammatory diseases, pathogenic axes, and targeted therapeutic strategies.

Diseases	Gene(s)	Pathogenic node	Shared phenotypes	Distinguishing features	Representative targeted therapies	Additional/escalation strategies
Type I interferonopathies						
Aicardi-Goutières syndrome (AGS1-7)	*TREX1* *RNASEH2A/B/C* *SAMHD1* *ADAR1* *IFIH1*	Self-nucleic-acid dysregulation/aberrant sensing of endogenous nucleic acids→ chronic IFN-I activation (51, 75)	Fever; rash; hematologic abnormalities	Chilblain-like rash; early-onset encephalopathy; intracranial calcifications; white-matter abnormalities; spasticity/dystonia	JAK inhibitors (baricitinib/ruxolitinib) (126, 127)	IFNAR1 blockade (anifrolumab); reverse-transcriptase inhibitors; IL-6 blockade (tocilizumab) (79, 83, 113)
STING-associated vasculopathy with onset in infancy (SAVI)	*STING1 (TMEM173)*	STING gain-of-function → constitutive STING signaling and persistent IFN-I activation (76, 120)	Fever; rash; systemic inflammation	Cold-sensitive acral ulceration/necrosis; facial erythema/telangiectasia; interstitial lung disease/pneumonitis	JAK inhibitors (ruxolitinib/baricitinib/tofacitinib) (76, 120, 122)	IFNAR1 blockade (anifrolumab); IL-6 blockade (tocilizumab) (116, 120)
Inflammasome-related disorders						
Familial Mediterranean fever (FMF)	*MEFV*	Pyrin inflammasome hyperactivation → excess IL-1 inflammation (55)	Recurrent fever; serositis; arthralgia	Erysipelas-like erythema; short self-limited attacks; secondary AA amyloidosis	IL-1 blockade (canakinumab/anakinra) (85, 86)	IL-6 blockade (tocilizumab); TNF inhibitors; JAK inhibitors (tofacitinib) (103, 138, 139)
Mevalonate kinase deficiency/hyper-IgD syndrome (MKD/HIDS)	*MVK*	Defective mevalonate/isoprenoid biosynthesis → impaired prenylation and enhanced IL-1β release (57, 58)	Recurrent fever; gastrointestinal inflammation; rash/arthralgia	Urinary mevalonic acid elevation; often elevated serum IgD	IL-1 blockade (canakinumab/anakinra) (58, 140)	IL-6 blockade (tocilizumab); TNF blockade (104, 105, 141, 142)
NLRC4-associated autoinflammatory disease (NLRC4-AID)	*NLRC4*	NLRC4 inflammasome hyperactivation → excess IL-18 and IL-1β release (61, 143)	Fever; enterocolitis; systemic inflammation	Recurrent or fulminant macrophage activation syndrome (MAS); marked hyperferritinemia; very high serum IL-18	IL-1 blockade (anakinra) (144, 145)	IL-18 blockade (IL-18BP); anti-IFN-γ (emapalumab) (62, 87)
NF-κB-centered disorders						
Blau syndrome	*NOD2*	Gain-of-function NOD2 signaling → NF-κB-driven inflammation (63, 64)	Arthritis; rash; systemic inflammation	Granulomatous dermatitis; boggy polyarthritis/tenosynovitis; chronic uveitis/panuveitis	TNF inhibitors (infliximab/adalimumab/etanercept) (146, 147)	JAK inhibitors (tofacitinib/baricitinib/upadacitinib); IL-1 blockade (canakinumab/anakinra) (118, 119, 147)
Haploinsufficiency of A20 (HA20)	*TNFAIP3*	A20 haploinsufficiency → impaired negative regulation of NF-κB signaling (65)	Recurrent fever; gastrointestinal inflammation; arthritis/rash	Recurrent oral and genital ulcers; early-onset Behçet-like disease pattern	TNF inhibitors (adalimumab/infliximab/etanercept); IL-1 blockade (canakinumab/anakinra) (96)	JAK inhibitors (tofacitinib/baricitinib/upadacitinib); IFNAR1 blockade (anifrolumab); IL-6 blockade (tocilizumab); B-cell-targeted therapies (belimumab, rituximab); PDE4 inhibitors (apremilast, roflumilast) (96)
NF-κB essential modulator-deleted exon 5 autoinflammatory syndrome (NEMO-NDAS)	*IKBKG*	IKBKG exon 5 skipping → NEMO-Δex5-mediated dysregulated NF-κB signaling (148)	Recurrent fever; rash systemic inflammation; hematologic abnormalities	Lobular panniculitis with subcutaneous nodules/lipoatrophy; uveitis; hepatitis/hepatosplenomegaly	TNF inhibitors (etanercept/adalimumab/infliximab) (149)	IL-6 blockade (tocilizumab); IL-1 blockade (anakinra) (149)

Representative diseases are grouped by major pathogenic axes, including type I interferonopathies, inflammasome-related disorders, and NF-κB-centered disorders. For each disease, the table summarizes the pathogenic node, overlapping clinical manifestations, distinguishing features, representative targeted therapies, and additional or escalation strategies reported in selected clinical contexts.

Inflammasome-related disorders illustrate a complementary principle: mechanistic relatedness does not translate into a single clinical form or a uniform downstream cytokine hierarchy. Inflammasome was defined in 2002 as a molecular platform for inflammatory caspase activation and pro-IL-1β processing (53). Unified by excessive IL-1β and/or IL-18 signaling, often coupled to pyroptotic inflammation, this category comprised 17 disease entities associated with 11 genes in the 2024 IUIS genotypic classification (21). Pyrin-related disorders centered on *MEFV* and *PSTPIP1* include FMF and PSTPIP1-associated inflammatory disease, spanning recurrent febrile serositis and acute-phase inflammation to more tissue-destructive phenotypes with sterile arthritis, inflammatory skin diseases and myositis (21, 54, 55). Mevalonate pathway defects involving *MVK* and *PMVK* underlie MKD/HIDS and PMVK deficiency, in which disturbed mevalonate metabolism converges on IL-1-driven periodic fever but with distinct accompanying features (21, 56–58). Canonical sensor defects involving *NLRP3*, *NLRP12*, *NLRC4* and *NLRP1* further illustrate genotype-weighted patterning, with relative enrichment of cold-triggered urticaria-like inflammation, enterocolitis and macrophage activation, or epithelial and cutaneous disease depending on the affected sensor (21, 59, 60). Even within this broad group, the clinically relevant inflammatory output may be more IL-1-dominant in some settings and more IL-18- or pyroptosis-linked in others (61, 62). Mechanistic classification therefore improves biological understanding, but it does not by itself determine which effector pathway is most therapeutically important in an individual patient.

The same complexity extends to non-inflammasome disorders involving NF-κB, ubiquitin, proteasome, and related signaling programs (21). NOD2-associated Blau syndrome exemplifies dysregulated proximal innate sensing, in which altered receptor autoinhibition and hyperactive downstream signaling drive granulomatous dermatitis, arthritis, and uveitis (63, 64). TNFAIP3 haploinsufficiency abrogates A20-mediated restraint of TNF-NF-κB signaling, leading to HA20, an early-onset Behçet-like disease marked by arthralgia, mucosal ulcers and ocular inflammation (26, 65). OTULIN is particularly instructive because different molecular consequences within the same gene can lead to distinct inflammatory or infectious phenotypes. Biallelic loss-of-function variants cause classical OTULIN-related autoinflammatory syndrome with early-onset recurrent fever, panniculitis or inflammatory skin lesions and diarrhea (21, 66–68), whereas dominant-negative heterozygous variants produce systemic inflammation through dysregulated linear ubiquitin signaling and TNF-related cell death (21, 69). By contrast, OTULIN haploinsufficiency primarily impairs cell-intrinsic defense against staphylococcal α-toxin in nonhematopoietic tissues, predisposing to severe infection-triggered necrosis of skin and lungs (70, 71). Conversely, syndromes such as PRAAS/CANDLE can arise from defects in multiple proteasome-related genes that converge on proteotoxic stress and chronic interferon induction, including *PSMB8*, *PSMG2*, *PSMB10*, *PSMB9*, *PSMB4* (72, 73). These observations show that molecular diagnosis is indispensable, but not sufficient.

## 3 Defined mechanisms, uncertain treatment

### 3.1 Targeted therapies based on gene-defined mechanisms

**Figure 2:**
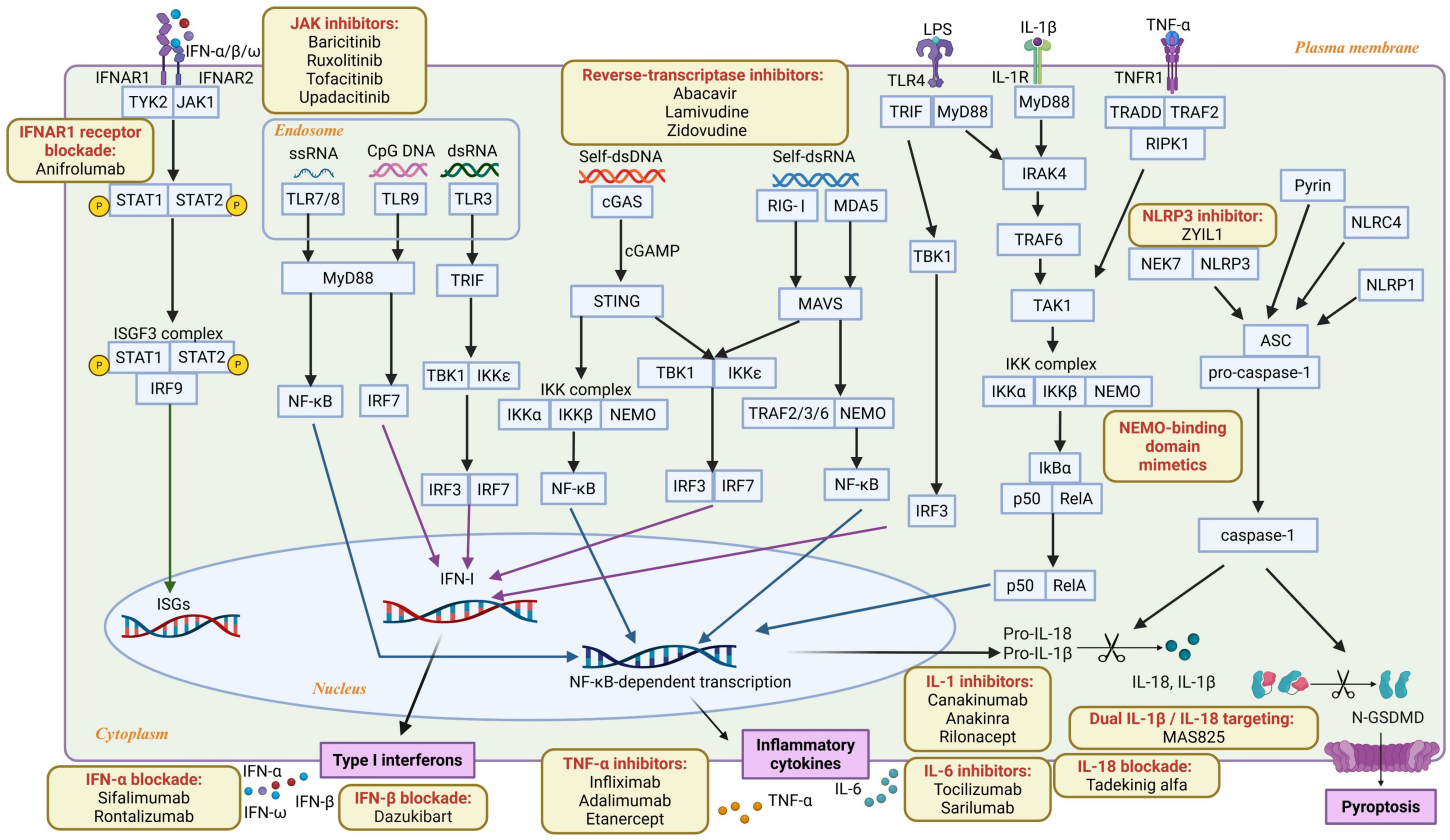
Representative targeted therapies across major inflammatory pathways in autoinflammatory diseases. Selected therapeutic targets are mapped across type I interferon, inflammasome, and NF-κB-related inflammatory pathways. Interventions are shown at different levels of inflammatory signaling, including upstream sensing, intracellular signal transduction, cytokine receptors, and downstream inflammatory mediators. This schematic illustrates that a single pathogenic axis may contain multiple therapeutic entry points, including JAK inhibition, interferon receptor blockade, IL-1 or IL-18 blockade, TNF inhibition, IL-6 receptor blockade, and other pathway-directed strategies. These approaches should not be regarded as clinically interchangeable, because treatment response is shaped by the dominant inflammatory module, organ involvement, disease stage, tissue reversibility, and treatment-limiting complications.

As pathogenic mechanisms have become clearer, treatment has shifted from empirical immunosuppression toward pathway-directed intervention. (**Figure 2**) In type I interferonopathies, distinct genetic defects converge on the IFN-I axis through endogenous nucleic acid accumulation, aberrant innate sensing, and impaired negative feedback (19, 27). Variants in *TREX1*, *RNASEH2A/B/C*, *SAMHD1*, and *ADAR1* enhance IFN-I induction through disturbed nucleic acid metabolism or sensing; *STING1* gain-of-function amplifies interferon output through constitutive signaling; and *USP18* or *ISG15* deficiency prolongs IFNAR signaling by impairing retro-control (19, 27, 51, 74–76). These lesions define several therapeutic entry points, including downstream JAK inhibition with ruxolitinib, baricitinib, upadacitinib and tofacitinib (77), receptor-level blockade with anti-IFNAR1 antibody anifrolumab (78, 79), ligand-level neutralization of circulating IFN-α with sifalimumab and rontalizumab (80, 81), or extracellular IFN-β with dazukibart (82), and more upstream attempts to reduce nucleic acid-driven interferon induction with abacavir, lamivudine, and zidovudine (83). These options illustrate how a shared pathway can yield several pharmacological entry points, but they should not be regarded as clinically equivalent.

A comparable logic applies to inflammasome-related disease, although the relevant downstream effector is not uniform across the spectrum (61, 62). *NLRP3*-associated CAPS, together with *MEFV*-associated FMF, *MVK*-associated MKD/HIDS, and *TNFRSF1A*-associated TRAPS, aligns more closely with an IL-1-centered inflammatory program, establishing IL-1 blockade with anakinra, canakinumab, and rilonacept as the core mechanism-based therapeutic strategy (9, 84–86). By contrast, *NLRC4*-associated disease and recurrent macrophage activation syndrome, marked by disproportionate IL-18 excess, have extended inflammasome-directed therapy toward IL-18 blockade, most notably with the recombinant IL-18 binding protein tadekinig alfa (87, 88). Dual targeting of IL-1β and IL-18 has further expanded this framework, exemplified by the bispecific monoclonal antibody MAS825 in recurrent or refractory macrophage activation syndrome (89, 90). More recently, this logic has also moved upstream, with selective NLRP3 inhibitors such as ZYIL1 entering early clinical development in CAPS, shifting inflammasome-directed therapy from downstream cytokine neutralization toward direct inhibition of the pathogenic sensor itself (91, 92).

Recognition of TNF-dependent inflammatory output in DADA2, OTULIN-related autoinflammatory syndrome, and selected cases of HA20 has similarly identified TNF as a therapeutically accessible node downstream of distinct defects (93–96). Monoclonal antibodies such as infliximab, adalimumab, and golimumab, the PEGylated Fab’ fragment certolizumab pegol, and the soluble TNF receptor fusion protein etanercept all intercept extracellular TNF and thereby prevent ligand-dependent TNFR signaling, with etanercept acting as a decoy receptor rather than an antibody (97). More proximal NF-κB-directed strategies remain largely experimental. Canonical NF-κB activation depends on IKK-complex signaling and NEMO-dependent pathway assembly (98). Experimental approaches have therefore attempted to inhibit this axis through NEMO-binding domain peptides, NBD mimetics, or selective IKKβ inhibitors (99, 100).

Additional targets further illustrate that therapy often intercepts actionable downstream amplifiers rather than the initiating lesion itself. IL-6 has emerged as a pharmacologically accessible inflammatory node, particularly in contexts marked by sustained acute-phase activation, amyloidogenic inflammation, or incomplete control with upstream cytokine blockade (101). Tocilizumab has been used most clearly in FMF, especially colchicine-resistant disease and AA amyloidosis, and more selectively in TRAPS and MKD/HIDS (101–105). Other targeted approaches include IL-36 blockade with spesolimab and imsidolimab in generalized pustular psoriasis and DITRA (106, 107), IL-12/23 or IL-17A inhibition in Behçet-like mucocutaneous and articular phenotypes (108, 109), apremilast for recurrent oral ulceration in Behçet syndrome (110), and RIPK1 inhibition as a proximal strategy linking inflammatory signaling and regulated cell death in disorders such as CRIA syndrome (111, 112). These advances show that gene-defined mechanisms can reveal rational therapeutic entry points, but do not establish a uniform hierarchy of actionable targets across diseases, tissues, or stages of inflammation.

### 3.2 Combined strategies beyond gene-defined mechanisms

Combined or sequential therapy is increasingly reported across monogenic autoinflammatory diseases, particularly in refractory, multisystem, or organ-threatening inflammation (113–115). This does not invalidate mechanistic reasoning; rather, it reflects the way inflammatory organization changes over time. Gene-defined lesions identify the point of disease initiation, whereas the clinically dominant effector network may later incorporate secondary cytokine circuits, organ-specific inflammatory layering, and shifting therapeutic dependencies. This pattern is particularly evident in type I interferonopathies. JAK inhibition remains the principal therapeutic strategy, yet residual inflammation may persist despite suppression of IFN-I signaling. In AGS7, prolonged ruxolitinib improved rash, hematologic abnormalities, and liver function, but systemic inflammation remained incompletely controlled until tocilizumab was added, indicating that IFNAR-JAK inhibition alone was insufficient to suppress all clinically relevant inflammatory circuitry (113). A similar signal is emerging from SAVI and CANDLE, in which anifrolumab provided additional benefit after prior JAK inhibition (115). In one refractory SAVI patient, both JAK inhibition and anti-IFN-β blockade failed to achieve durable control, whereas subsequent treatment with anifrolumab plus tocilizumab improved vasculitic lesions and enabled treatment de-escalation (116).

Inflammasome-related disease reveals a similar but not identical pattern. Once inflammation becomes clinically layered, suppression of a single canonical cytokine axis may no longer suffice. In NLRP1-associated autoinflammation, a severe inflammatory attack required anakinra plus ruxolitinib, with complete remission maintained at three years, suggesting that clinically expressed inflammation was not fully captured by an isolated IL-1-centered process (114). In life-threatening NLRC4-associated hyperinflammation, sequential attempts that included IL-1 blockade, TNF-α blockade, and α4β7-integrin inhibition failed before sustained control was achieved with IL-18 inhibition, underscoring that mechanistic definition does not by itself identify the most effective downstream target (87). PAAND further shows this disconnect, as anakinra was not superior to TNF-α-targeting biologics despite direct implication of IL-1β dysregulation (117). Different effector programs may therefore assume pathogenic priority at different stages, supporting combined therapy when parallel inflammatory outputs remain active and sequential retargeting when the dominant sustaining pathway changes over time. Comparable complexity extends beyond interferonopathies and inflammasome disease. In Blau syndrome, tofacitinib improved arthritis, uveitis, and rash only after prior failure of methotrexate, tocilizumab, and etanercept (118). Subsequent maintenance with baricitinib in refractory disease further illustrates the need for sequential adjustment rather than simple mechanism-to-drug matching (119). Across these settings, clinically expressed inflammation is often organized by interacting effector modules rather than a single dominant node. Combined or sequential therapeutic strategies should therefore be understood not as failures of mechanistic medicine, but as evidence that the circuitry sustaining disease may diverge from the initiating lesion during disease evolution.

## 4 Rational therapies, variable responses

### 4.1 Intra-disease variability in therapeutic responses

Therapeutic complexity is further illustrated by the marked response variability within the same monogenic disease. In SAVI, a familial series dominated by interstitial lung disease documented poor response to ruxolitinib, whereas the accompanying literature review showed markedly divergent outcomes among ruxolitinib-treated cases, ranging from pulmonary and cutaneous improvement to transient benefit, relapse, or continued progression of interstitial lung disease (120, 121). Even within a single STING1-associated disorder, responsiveness to the same agent is distinctly non-uniform. Heterogeneity extends to individual JAK inhibitors. In a 2-year follow-up case, inadequate control with tofacitinib was followed by broader clinical improvement after substitution with ruxolitinib, suggesting that therapeutic responsiveness is not fully interchangeable and may require iterative adjustment (122). This divergence is mechanistically plausible because ruxolitinib and tofacitinib impose different constraints on interferon-linked inflammatory signaling through different JAK selectivity profiles (JAK1/JAK2 and JAK1/JAK3, respectively) (123). A comparable pattern is evident in AGS. Ruxolitinib in an ADAR1-related AGS6 case reduced interferon signature but yielded limited neurological improvement (124), whereas baricitinib in an RNASEH2B-related AGS2 case was associated with markedly greater recovery of cognitive, communicative, and relational function (125). Within the same clinical syndrome, treatment response therefore depends not only on pathway matching, but also on timing of intervention and tissue reversibility, particularly in the central nervous system (126, 127).

Comparable intra-disease heterogeneity extends beyond interferonopathies. In DADA2, patients with predominantly vasculitic or inflammatory phenotypes often respond well to TNF inhibition, and individual cases have also shown clinical improvement with adalimumab-based therapy (128, 129). By contrast, in patients with bone marrow failure, refractory cytopenias, or severe hematologic disease, anti-cytokine treatment is often insufficient, and hematopoietic stem cell transplantation may represent the preferred definitive approach (128, 130, 131). CAPS illustrates a related form of variability under canonical IL-1 blockade. In a canakinumab-treated series, some patients achieved sustained disease control on standard regimens, whereas others required dose escalation or interval shortening, particularly in more severe phenotypes (132, 133). Longitudinal auditory follow-up under anti-IL-1 therapy likewise demonstrated non-uniform trajectories, with stabilization in most patients, improvement in some, and progression in a subset requiring therapeutic escalation (134). Blau syndrome reinforces the same principle: refractory disease has improved with tofacitinib after failure of methotrexate, tocilizumab and etanercept, while subsequent maintenance with baricitinib indicates that even within a single NOD2-associated disorder, the most effective targeted agent may not remain constant across patients or phases (118, 119). PAAND further shows that even within a single inflammasome-related disorder, mechanistically matched therapy may yield heterogeneous responses, as anakinra produced inconsistent benefit and was not superior to TNF-α-targeting biologics (117). These observations indicate that intra-disease therapeutic heterogeneity is structured rather than stochastic. Within the same monogenic disorder, treatment response is shaped by allele effect, dominant organ phenotype, disease stage, tissue reversibility, and the persistence of parallel effector programs beyond the initiating lesion.

### 4.2 Toward a clinically usable therapeutic framework

Molecular diagnosis remains indispensable because it defines the initiating lesion and narrows the relevant pathogenic axis (9, 19, 27). However, genotype alone does not determine which inflammatory node is currently dominant, which organ system is at greatest immediate risk, or which therapeutic dependency is most actionable in an individual patient (17–19, 27). Treatment in autoinflammation should therefore be understood as genotype-informed rather than genotype-determined. A clinically usable framework for treatment selection, escalation, and sequencing should incorporate at least five variables. First, the dominant inflammatory module must be defined at the time of decision-making. Although the initiating lesion may reside in the IFN-I, inflammasome, TNF, or NF-κB axis, established disease may instead be sustained by secondary cytokine circuits or parallel effector programs not fully extinguished by blocking the original pathway (101, 113, 114). Second, the dominant organ phenotype should guide prioritization, as the same molecular diagnosis may require different therapeutic logic depending on whether the decisive burden lies in interstitial lung disease, central nervous system involvement, vasculopathy, intestinal inflammation, or hematologic failure (38, 42, 120). Third, disease stage matters: early disease may remain largely pathway-dependent and more amenable to targeted suppression, whereas later disease may reflect inflammatory layering, cumulative damage, or partial uncoupling from the original lesion (38, 113, 114, 120). Fourth, therapeutic reasoning must account for tissue reversibility, because suppression of inflammatory signaling does not ensure functional recovery once irreversible structural injury has occurred, particularly in the lung, vasculature, auditory system, or central nervous system (38, 120, 124, 134). Fifth, treatment-limiting complications and competing risks must be considered. Infectious vulnerability, cytopenias, or hepatic abnormalities may reflect disease biology, treatment toxicity, or both (17, 18, 135).

These variables have direct therapeutic implications. Single-node targeting is most likely to succeed when the dominant effector remains closely aligned with the initiating lesion and clinically relevant tissue injury is still reversible (17, 18). Combined therapy becomes more rational when parallel inflammatory outputs remain active, whereas sequential retargeting is more appropriate when therapeutic dependency diversifies over time (87, 113–119). In selected settings, especially when severe hematologic dysfunction, marrow failure, or advanced organ-threatening disease predominates, definitive interventions such as hematopoietic stem cell transplantation may be more appropriate than repeated escalation of cytokine-directed therapy alone (136, 137). Current evidence remains weighted toward case reports, small series, and pathway-level extrapolation rather than comparative or prospective studies, so treatment choice remains probabilistic rather than deterministic. Even so, a framework that is genotype-informed, phenotype-weighted, organ-prioritized, and time-sensitive may better align mechanistic insight with real-world clinical decision-making in autoinflammation.

## 5 Discussion and future perspectives

Autoinflammation has become mechanistically tractable, but therapeutic predictability has not advanced in parallel. Monogenic autoinflammatory diseases make this disconnect especially clear: the initiating lesion, the sustaining inflammatory circuitry, and the process currently driving tissue injury may diverge over time. This helps explain why pathway-matched therapy may yield incomplete benefit, why combined or sequential strategies are often required, and why responses vary substantially even within the same genetic disorder. These disorders therefore do not conform to a simple gene-to-pathway-to-therapy model.

Despite major therapeutic advances, currently available targeted therapies remain limited by incomplete responses, secondary loss of efficacy, dose escalation, treatment switching, and the frequent need for prolonged or combined immunomodulation in refractory or organ-threatening disease. Long-term safety remains incompletely defined, particularly in children, and treatment-related complications such as infection risk, cytopenias, hepatic abnormalities, impaired vaccine responses, and cumulative immunosuppression may overlap with disease-intrinsic immune dysfunction. Moreover, suppression of inflammatory signaling does not necessarily reverse established tissue damage, especially in the central nervous system, lung, vasculature, and auditory system. These limitations are compounded by an evidence base still dominated by case reports, small cohorts, retrospective series, and heterogeneous treatment experience. Future progress will require biomarker-guided and longitudinally adaptive therapeutic frameworks that integrate genotype, pathway activity, cytokine or interferon signatures, organ-specific outcomes, disease stage, and treatment exposure. Prospective registries and longitudinal cohort studies linking molecular signatures, therapeutic interventions, safety outcomes, and organ-specific trajectories will be essential for translating mechanistic insight into clinically usable precision medicine in autoinflammation.
